# A Practical Treatise on the Diagnosis, Pathology, and Treatment of the Diseases of the Heart

**Published:** 1859-11

**Authors:** 


ART. VIII.—*A Practical Treatise on the Diagnosis, Pathology, and Treatment of the Diseases of the Heart.* By AUSTIN FLINT, M.D., Professor of Clinical Medicine in the New Orleans School of Medicine; Physician to the New Orleans Charity Hospital, etc., etc. 8vo., pp. 473. Philadelphia: Blanchard & Lea, 1859.

THE treatise of Professor Flint reached us too late to permit us to do anything more than simply to announce its publication. An elaborate notice, from the pen of one of our most able and learned collaborators, will appear in our next issue. A cursory examination of the work satisfies us that it is a highly meritorious production, destined to secure a permanent place in the medical literature of the country.

